# No long-term effect of past *Pneumocystis jirovecii* pneumonia on pulmonary function in people with HIV

**DOI:** 10.1097/QAD.0000000000003540

**Published:** 2023-03-09

**Authors:** Patrick G.A. Oomen, Inez Bronsveld, Andy I.M. Hoepelman, Berend J. van Welzen, Tania Mudrikova

**Affiliations:** a Department of Internal Medicine; b Department of Pulmonary Diseases, University Medical Centre Utrecht, Utrecht, The Netherlands.

**Keywords:** diffusion capacity, HIV, *Pneumocystis jirovecii* pneumonia, pulmonary function, transfer factor for carbon monoxide

## Abstract

To assess the impact of past *Pneumocystis jirovecii* pneumonia (PJP) on the pulmonary diffusion capacity in people with HIV (PWH) with a history of advanced immunodeficiency.

**Design:**

Prospective cross-sectional study.

**Methods:**

Adult PWH with past PJP >1 year ago were included as the study group. The control group consisted of PWH with a nadir CD4^+^ lymphocyte count <200 cells/mm^3^, matched by age, sex, smoking status and time since HIV diagnosis. All PWH completed a pulmonary function test (PFT) consisting of pre-bronchodilation spirometry, body plethysmography and single-breath carbon monoxide transfer factor (TLCO) measurement. TLCO, diffusion impairment (defined as a TLCO *Z*-score <−1.645), total lung capacity (TLC) and forced expiratory volume in one second/forced vital capacity (FEV1/FVC) *Z*-scores were assessed. Multivariable regression analyses were conducted with *Z*-scores and odds of diffusion impairment as outcomes.

**Results:**

PFTs of 102 participants were analyzed, 51 of whom had past PJP with a median of 10 years since PJP. Mean TLCO *Z*-score and diffusion impairment rate did not differ significantly between groups (*P* = 0.790; *P* = 0.650). Past PJP was not independently associated with TLCO *Z*-score [*β* = 0.14; 95% confidence interval (CI) −0.30–0.57], diffusion impairment (odds ratio 1.00; 95% CI 0.36–2.75) nor TLC or FEV1/FVC *Z*-scores, whereas current (vs. never) smoking was associated with more diffusion impairment and lower TLCO *Z*-scores.

**Conclusion:**

In our study, past PJP was not associated with long-term diffusion impairment. Our findings suggest that smoking plays a more important role in persistent pulmonary function impairment whereas PJP-related changes seem to be reversible.

## Introduction

*Pneumocystis jirovecii* pneumonia (PJP) is one of the most common opportunistic infections in people with HIV (PWH) and advanced immunodeficiency [1], leading to severe hypoxemia due to diffusion capacity impairment [2]. The introduction of combination antiretroviral therapy and improved PJP treatment strategies have led to a significant decline in mortality from PJP, but it is unclear whether pulmonary function abnormalities completely resolve in this population [3,4]. Older, short-term data suggest that diffusion impairment persists in a significant percentage of PWH [5–8], but the long-term consequences of past PJP remain largely unknown.

Establishing the potential long-term impact of PJP on pulmonary function, and more specifically the diffusion capacity, is clinically relevant, as the background prevalence of diffusion impairment in PWH is high, likely due to prevalent smoking and HIV infection itself [9–13]. In this study, we evaluated the pulmonary function in PWH with previous advanced immunodeficiency with and without past PJP.

## Methods

### Study design and population

We performed a prospective, single-center, cross-sectional analysis of pulmonary function in adult PWH with a history of advanced immunodeficiency (defined as a nadir CD4^+^ lymphocyte count <200 cells/mm^3^), all under follow-up at the University Medical Center Utrecht (UMCU), the Netherlands. We chose to include only PWH with a history of advanced immunodeficiency as control group as earlier research identified lower CD4^+^ cell count as predictors for diffusion impairment, possibly due to damage caused by HIV-associated immune activation or not clinically apparent opportunistic pulmonary infections [10,13]. The study group consisted of PWH with a history of PJP >1 year ago; PWH in the control group had no previous PJP. Both groups were matched regarding age, sex at birth, smoking status and time since HIV diagnosis to limit confounding. All participants underwent standardized pulmonary function testing. To ensure we did not miss pulmonary impairment from PJP that was misclassified as chronic obstructive pulmonary disease (COPD) due to smoking, COPD was deliberately not chosen as an exclusion criterion. Exclusion occurred in case of active pulmonary infection, cardiac decompensation, onset of unexplained dyspnea, tachypnea or other respiratory complaints within three weeks before pulmonary function test (PFT) and preexistent conditions unrelated to PJP but known to impact pulmonary function impairment (i.e., interstitial pulmonary disease, pulmonary hypertension or prior tuberculosis). Demographic, clinical and biochemical data at the time of PFT were collected from electronic medical records, with certain parameters (e.g., smoking habits) specifically asked.

The study was approved by the UMCU ethical review board and informed consent was obtained from all participants. The study was funded by Gilead Sciences, which had no role in trial design, data collection, analysis or manuscript preparation.

### Pulmonary function testing

Each participant performed one PFT consisting of pre-bronchodilation spirometry, body plethysmography and single-breath transfer factor for carbon monoxide (TLCO) measurement (i.e., measurement of diffusion capacity) according to European Respiratory Society/American Thoracic Society (ERS/ATS) standards using a Geratherm spirometer [14–17]. If no ERS/ATS-qualifying effort was recorded, the PFT was excluded.

The TLCO *Z*-score was chosen as primary outcome. As secondary outcome we assessed diffusion impairment defined as a TLCO *Z*-score <−1.645 (i.e., belonging to the <5th percentile), with a *Z*-score of −1.65 to −2.50, −2.51 to −4.00, <−4.10 defined as mild, moderate and severe, respectively [17]. Other secondary outcomes - obstructive and restrictive impairment - were defined as a forced expiratory volume in one second/forced vital capacity (FEV1/FVC) and total lung capacity (TLC) *Z*-score <−1.645. Global Lung Initiative reference value *Z*-scores based on age, sex at birth, height and, for spirometry, ethnicity were used and TLCO values were corrected for hemoglobin [14–16]. All PFTs were reviewed by a pulmonologist and participants with a successful TLCO measurement were included in the analysis.

### Statistical analysis

Continuous data were expressed as mean ± standard deviation or median and interquartile range for parametric and nonparametric data. A chi-square and Mann–Whitney U or independent samples *t*-tests were used to compare categorical and continuous variables. Multivariable linear regression and logistic regression using Firth's bias correction for small samples were used to assess mean *Z*-scores and pulmonary impairment [18]. Additionally, in PWH with PJP the association between time since PJP, adjunctive steroid-use during PJP and TLCO *Z*-score was investigated. Multicollinearity and assumptions of linearity, homoscedasticity and normality of residuals were assessed. Two sensitivity analyses were performed using only PWH with an undetectable viral load at the time of PFT and without previous COVID-19**.** A two-sided alpha level of 0.05 was used and all statistical analyses were conducted using RStudio (v1.3.1093).

### Sample size calculation

We hypothesized that past PJP would result in lower TLCO *Z*-scores and calculated that a sample of 100 participants would provide our study with 80% power to show a significant effect of PJP, with an estimated TLCO *Z*-score difference between study groups of 0.57.

## Results

### Demographics

A total of 105 PWH underwent the PFT evaluation between 2016 and 2022. Three of them were excluded as their PFT did not meet ERS/ATS quality standards. This left 102 participants available for analysis, including 51 with past PJP with median 10 years since PJP [interquartile range (IQR) 5.00–16.00]. Six of all participants had a mild COVID-19 infection more than 6 months before PFT; none of them required specific treatment or hospital admission. Both groups were comparable regarding age (median 54.00 vs. 53.00 years, *P* = 0.453) and sex at birth (44/51 (86.27%) vs. 45/51 (88.24%), *P* = 0.767). Compared to those without PJP (PJP-), PWH with PJP (PJP+) had lower median nadir CD4^+^ lymphocyte counts (28.00 vs. 78.00 cells/mm^3^, *P* < 0.001) (Table 1).

**Table 1:** Characteristics of PWH according to PJP status at time of PFT.

Demographics	PJP+		PJP−		*P*-value
	*n* = 51	(IQR) (%)	*n* = 51	(IQR) (%)	
Age (years)	54.00	50.00–58.00	53.00	46.00–59.00	0.453
Sex at birth (male)	44	86.27	45	88.24	0.767
Clinical characteristics					
Time since HIV diagnosis (years)	10.00	6.00–17.00	11.00	7.00–17.00	0.245
Time since PJP (years)	10.00	5.00–16.00	-	-	-
Time since start antiretroviral therapy (years)	10.00	6.00–16.00	10.00	7.00–16.00	0.341
Smoking					0.834
- current	27	52.94	24	47.06	
- former	13	25.49	15	29.41	
- never	11	21.57	12	23.53	
Mode of transmission					0.207
- MSM	25	49.02	31	60.78	
- heterosexual	8	15.69	10	19.61	
- other/unknown	18	35.29	10	19.61	
History of pneumothorax ^a^	5	9.80	-	-	
Admission to ICU during PJP ^a^	9	17.64	-	-	
Adjunctive steroids during PJP ^a^	33	64.71	-	-	
Biochemical characteristics					
Nadir CD4^+^ lymphocyte count (cells/mm^3^)	28.00	10.00–50.00	78.00	41.00–152.00	<0.001
CD4^+^ lymphocyte count at PFT^a^	478.00	372.75–559.75	537.00	392.50–691.75	0.120
VL <400 at PFT (cop/ml)^a^	51	100	51	100	-

All categorical data are expressed as frequency (percentage) and all continuous data are expressed as median (interquartile range). a. Missing data: CD4^+^ lymphocyte count at PFT (one PJP− (1.96%)/one PJP+ (1.96%)), History of pneumothorax (3 PJP+ 5.88%), Admission to ICU during PJP (3 PJP+ 5.88%), Adjunctive steroids during PJP (2 PJP+ 3.92%).

a Detectable viral loads at the time of PFT were observed for three PJP+ (VLs of 319, 83, 75 copies/ml) and one PJP- (VL of 179 copies/ml). ICU, intensive care unit; IQR, interquartile range; MSM, men who have sex with men; PFT, pulmonary function test; PJP, *Pneumocystis jirovecii* pneumonia; PWH, people with HIV; VL, viral load.

### Diffusion impairment

TLCO *Z*-score as diffusion parameter was found to be similar between PJP+ and PJP− (−0.98 (1.11) vs. −0.92 (1.04), *P* = 0.790). Multivariable linear regression showed that only current (*vs.* never) smoking was associated with lower TLCO *Z*-scores (*β* −1.10; 95% CI −1.61 to −0.59), while no association was observed with past PJP (*β* 0.14; 95% CI −0.30–0.57) (Table 2).

**Table 2:** Multivariable logistic and linear regression analysis for TLCO *Z*-scores and diffusion impairment (defined as TLCO Z-score <-1.645).

	TLCO *Z*-score			Diffusion impairment		
	*β*	95% CI	*P*-value	OR	95% CI	*P*-value
Past PJP (vs. no past PJP)	0.14	−0.30–0.57	0.545	1.00	0.36–2.75	0.997
Age at PFT (per year increase)	−0.02	−0.04–0.01	0.164	1.00	0.95–1.06	0.971
Male sex (vs. female sex)	0.21	−0.40–0.81	0.504	0.46	0.13–1.65	0.235
Time since HIV diagnosis (per year increase)	0.01	−0.02–0.04	0.454	1.02	0.95–1.09	0.648
Smoking						
- never	1		−	1		−
- former	−0.14	−0.63–0.36	0.597	1.68	0.51–5.74	0.396
- current	−1.10	−1.61 to −0.59	<0.001	6.02	1.94–18.72	0.002
Nadir CD4^+^ lymphocyte count (per 5 cell/mm^3^ increase)	0.02	0.00–0.04	0.061	0.97	0.93–1.02	0.195

CI, confidence interval; OR, odds ratio; PFT, pulmonary function test; PJP, *Pneumocystis jirovecii* pneumonia; TLCO, transfer factor for carbon monoxide.

When diffusion impairment was defined dichotomously as TLCO *Z*-score <−1.645, comparable rates were found in PJP+ and PJP− (14/51 (27.45%) vs. 12/51 (24.53%), *P* = 0.650). Rates of mild and moderate diffusion impairment were also similar between groups and no severe diffusion impairment was observed. Current (vs. never) smokers had higher odds of diffusion impairment [odds ratio (OR) 6.02; 95% CI: 1.94–18.72], whereas similar odds were found for past PJP vs. no past PJP (OR 1.00; 95% CI: 0.36–2.75). See Table S1, Supplemental Digital Content 1, for all PFT Z-scores, % predicted and exact rates of diffusion impairment severity by group.

The association between time since PJP, steroid-use during PJP and TLCO *Z*-score was evaluated in the PJP+ group. Only current smoking was independently associated with lower TLCO *Z*-scores (*β* −1.36; 95% CI −2.10 to −0.63) and no association was found with time since PJP (*β* 0.03; 95% CI −0.02–0.08) nor steroid-use during PJP (*β*−0.60; 95% CI −1.21–0.00).

### Obstructive and restrictive impairment

FEV1/FVC Z-scores were similar for PJP+ and PJP− (−0.31 (1.08) vs. −0.28 (1.10), *P* = 0.894). No independent association for past PJP was found (*β* 0.03; 95% CI −0.44–0.50), whereas current smoking was associated with lower FEV1/FVC *Z*-scores (*β* −0.69; 95% CI −1.24 to −0.14). Past PJP was not associated with obstructive impairment (4/51 (7.84%) vs. 6/51 (12.00%), *P* = 0.484). See Table S2, Supplemental Digital Content 2, for linear regression results.

Similar TLC *Z*-scores were found for both groups (−0.09 (1.04) vs. –0.01 (1.13), *P* = 0.705). Current smoking was independently associated with higher TLC *Z*-scores (*β* 0.58; 95% CI 0.04–1.12). No association was found for past PJP (*β* 0.09; 95% CI −0.36–0.55). Restrictive impairment was not associated with past PJP (4/51 (7.84%) vs. 2/51 (3.92%), *P* = 0.400). Given the low number of obstructive and restrictive impairment, multivariable logistic regression was not performed for these outcomes.

### Sensitivity analyses

Sensitivity analyses were conducted using 98/102 PWH with an undetectable viral load at the time of PFT (excluding 3/51 PJP+ and 1/51 PJP−) and 96 without previous COVID-19 (excluding 6/51 PJP+). All results were similar to those of the main analyses.

## Discussion

In this cross-sectional study on the potential long-term pulmonary sequelae of PJP in PWH, we found no effect of past PJP on the diffusion capacity, evaluated either as a continuous TLCO *Z*-score or dichotomously as *Z*-score <−1.645. Current (vs. never) smoking was found independently associated with lower TLCO *Z*-scores and diffusion impairment. Notably, more than 25% of PWH in our study had diffusion impairment, as defined by the latest ERS/ATS guidelines [14–17].

Previous PJP does not seem to put PWH at greater risk of long-term diffusion impairment. Although their diffusion capacity is substantially more often diminished compared to the general population, this seems to be driven by other factors, such as HIV infection and smoking [9–13]. Though the effect of HIV infection itself could not be investigated with our study design, the presented findings confirm the detrimental effect of smoking. Other authors who specifically investigated PJP's effect on diffusion impairment reported conflicting results, but their studies had short follow-up times or did not specify the time since PJP – the latter being particularly relevant given the improvement of pulmonary function over time described in some studies [5–8]. To our knowledge, this is the first study which examined long-term sequelae of past PJP, with a median of 10 years since PJP.

We hypothesize that PJP-related damage either recovers in the long-term or its contribution is negligible in the presence of persistent pulmonary impairment from smoking and HIV infection. From a pathophysiological perspective, the diffusion impairment is the result of a different process in the acute and postacute phase of PJP. It is assumed that high influx of inflammatory cells is responsible for hypoxemia in the acute stage of PJP [2], whereas postacute diffusion impairment is suggested to be due to lasting alveolar surfactant alterations resulting from the inflammatory response [19,20]. Our data suggest that the latter is probably reversible over time. A gradual improvement of the pulmonary function after PJP is supported by the findings of a longitudinal study of 84 PJP patients using HR-CT scans [21], in which complete resolution of the radiological abnormalities was seen in 90% of participants 302 days after PJP. It should be noted, however, that this study did not include PWH, but people on immunosuppressive therapy.

Our study has several strengths. Next to the aforementioned long-term PJP-related outcomes, the PFT measurement was performed systematically in accordance with the latest ERS/ATS quality standards and a matched group of PWH with advanced immunodeficiency without past PJP serving as control, as well as multivariable adjustment were used to minimize confounding bias.

Certain limitations also apply to our study. We could not account for previous bacterial pneumonias or underlying undiagnosed pulmonary vascular and interstitial lung disease, all of which can result in diffusion impairment, but given the relatively homogeneous study population, we do not expect these factors to differ between groups [22]. Additionally, the observed association between PJP and pulmonary function impairment may be an underestimation of the true association, since PWH who died due to severe PJP-sequelae with potentially severe impairment were not included in our study. Future research should preferably be longitudinal in nature and include the above factors, to ultimately disentangle the effects of specific risk factors on the course of pulmonary impairment in PWH.

In conclusion, our study did not show an association between past PJP and persistent diffusion impairment in PWH. Our findings suggest that PJP-related pulmonary damage recovers in the long-term or that its contribution, in the presence of pulmonary impairment from smoking or HIV infection, is marginal.

## Acknowledgements

Author contributions: I.B., B.W. and T.M. designed the study. B.W. wrote the study protocol. P.O. was responsible for the site work including the recruitment and data collection. All authors had access to data. P.O. performed the analysis, interpreted results and drafted the manuscript. All authors contributed to the interpretation of the data, critically reviewed the manuscript and approved the final manuscript.

Funding: The study was funded by Gilead Sciences, which had no role in trial design, data collection, analysis or manuscript preparation.

### Conflicts of interest

There are no conflicts of interest.

## Supplementary Material

Supplemental Digital Content

## Supplementary Material

Supplemental Digital Content
