# Supplementary material for: No long-term effect of past Pneumocystis jirovecii pneumonia on pulmonary function in people with HIV
Source: AIDS. 2023 Mar 9;37(8):1263–7. doi: 10.1097/QAD.0000000000003540 (PMC10241413; doi:10.1097/QAD.0000000000003540)
Supplement: Supplemental Digital Content [file aids-37-1263-s001.docx]

**Supplemental Digital Content 1**

**Table S1.** Pulmonary function test results according to PJP-status

|  | PJP+ | | PJP- | |  | |
| --- | --- | --- | --- | --- | --- | --- |
| Diffusion indices * | n = 51 | (SD) (%) | n = 51 | (SD) / (%) | | *p-value* |
| KCO (Z-score) | -0.90 | 1.12 | -0.80 | 1.20 | | 0.642 |
| KCO (% predicted) | 87.56 | 15.66 | 89.06 | 16.73 | | 0.641 |
| TLCO (Z-score) | -0.98 | 1.11 | -0.92 | 1.04 | | 0.790 |
| TLCO (% predicted) | 86.08 | 15.71 | 86.08 | 15.71 | | 0.772 |
| Diffusion Impairment (TLCO Z-score <-1.645) | 14 | 27.45 | 12 | 24.53 | | 0.650 |
| Severity of diffusion impairment   - mild (Z-score -1.65 – -2.50) - moderate (Z-score -2.51 – 4.00) - severe (Z-score <-4.10) | 8  6  0 | 57.14  42.86  0 | 8 4  0 | 66.66  33.33  0 | | 0.619 |
| Obstruction indices ^a^ |  |  |  |  | |  |
| FVC (Z-score) | 0.07 | 1.01 | -0.02 | 1.05 | | 0.675 |
| FVC (% predicted) | 100.97 | 13.91 | 99.95 | 14.67 | | 0.721 |
| FEV1 (Z-score) | -0.13 | 1.07 | -0.12 | 1.30 | | 0.961 |
| FEV1 (% predicted) | 98.01 | 14.70 | 97.23 | 17.26 | | 0.807 |
| FEV1/FVC (Z-score) | -0.31 | 1.08 | -0.28 | 1.10 | | 0.894 |
| FEV1/FVC (% predicted) | 97.35 | 8.88 | 96.96 | 10.32 | | 0.838 |
| Obstructive Impairment (FEV1/FVC Z-score <-1.645) | 4 | 7.84 | 6 | 12.00 | | 0.484 |
| Restriction indices |  |  |  |  | |  |
| TLC (Z-score) | -0.09 | 1.04 | -0.01 | 1.13 | | 0.705 |
| TLC (% predicted) | 98.92 | 12.43 | 100.13 | 13.61 | | 0.641 |
| Restrictive Impairment (TLC Z-score <-1.645) | 4 | 7.84 | 2 | 3.92 | | 0.400 |

All categorical data are expressed as frequency (percentage) and all continuous data are expressed as mean (standard deviation).
a. Spirometry data of one PJP- is missing due to not meeting ERS/ATS-qualifying standards.

***** Diffusion values are corrected for hemoglobin.

Abbreviations: FEV1, forced expiratory volume in 1 second; FVC, forced vital capacity; KCO, transfer coefficient for carbon monoxide; SD, standard deviation; TLC, total lung capacity; TLCO**,** transfer factor for carbon monoxide**;**
