# Supplementary material for: No long-term effect of past Pneumocystis jirovecii pneumonia on pulmonary function in people with HIV
Source: AIDS. 2023 Mar 9;37(8):1263–7. doi: 10.1097/QAD.0000000000003540 (PMC10241413; doi:10.1097/QAD.0000000000003540)
Supplement: Supplemental Digital Content [file aids-37-1263-s002.docx]

**Supplemental Digital Content 2**

**Table S2.** Multivariable linear regression analysis for FEV1/FVC and TLC Z-scores

|  | FEV1/FVC Z-score | | | TLC Z-score | | |  |
| --- | --- | --- | --- | --- | --- | --- | --- |
|  | ***β*** | **95% CI** | ***p-value*** | ***β*** | **95% CI** | ***p-value*** | |
| Past PJP (*vs.* no past PJP) | 0.03 | -0.44 – 0.50 | 0.904 | 0.09 | -0.36 – 0.55 | 0.689 | |
| Age at PFT (per year increase) | 0.00 | -0.02 – 0.02 | 0.964 | 0.01 | -0.02 – 0.03 | 0.545 | |
| Male sex (*vs.* female sex) | 0.11 | -0.54 – 0.75 | 0.751 | 0.15 | -0.48 – 0.77 | 0.651 | |
| Time since HIV diagnosis (per year increase) | 0.00 | -0.03 – 0.04 | 0.786 | 0.01 | -0.02 – 0.04 | 0.528 | |
| Smoking  - never  - former  - current | 1  0.11  -0.69 | -0.44 – 0.65  -1.24 – -0.14 | -  0.704  **0.016** | 1  -0.01  0.58 | -0.54 – 0.51  0.04 – 1.12 | -  0.963  **0.037** | |
| Nadir CD4^+^ lymphocyte count (per 5 cell/mm^3^ increase) | 0.01 | -0.01 – 0.03 | 0.515 | 0.02 | 0.00 – 0.04 | 0.092 | |

Abbreviations: FEV1/FVC, forced expiratory volume in one second/ forced vital capacity; PJP, *Pneumocystis jirovecii* Pneumonia; PFT, pulmonary function test, TLC, total lung capacity.
